# Olanzapine enhances the effect of conventional drugs in chemotherapy inducing nausea and vomiting: A randomized clinical trial

**DOI:** 10.22088/cjim.13.2.6

**Published:** 2022

**Authors:** Atefe Khani, Ali Eishy Oskuyi, Rahim Asghari, Hamid Reza Khalkhli, Hamdollah Sharifi

**Affiliations:** 1 Department of Pharmacology, Pharmacy Faculty, Urmia University of Medical Sciences, Urmia, Iran; 2 Department of Internal Medicine, Faculty of Medicine, Urmia University of Medical Sciences, Urmia-Iran; 3 Department of Biostatistics and Epidemiology, School of Medicine; 4 Clinical Research Development Unit of Imam Khomeini Hospital, Urmia University of Medical Sciences, Urmia, Iran

**Keywords:** Olanzapine, chemotherapy-induced nausea and vomiting (CINV), chemotherapy, cancer

## Abstract

**Background:**

Chemotherapy inducing nausea and vomiting (CINV) is one of the significant side effects of anti-cancer treatment, and its full prevention is a potential challenge. This study was done to specify the effect of olanzapine in this setting.

**Methods:**

In this randomized, double-blind, clinical trial study, olanzapine was compared with a placebo in combination with dexamethasone and granisetrone in patients with cancer. Patients in the intervention group received dexamethasone , granisetron and olanzapine. Patients in the control group received a placebo instead of olanzapine. Overall, acute nausea and vomiting prevention were the primary and secondary end points; complete response (no nausea,no vomiting) in the delayed period of chemotherapy was the third end point. Response to treatment was evaluated by the Functional Living Index Emesis (FLIE) questionnaire completion in the first, the third and the fifth of chemotherapy.

**Results:**

Percentage reduction in mean±SD nausea and vomiting in the overall phase (0-120 hours) of intervention group compared to the control group respectively were 29.94±2.06, 69.75±2.32 [(57.93% reduction (p<0.001)]. For the acute phase (0-24 hours) were 26.08±2.36, 51.85±2.24 [(47.21% reduction (p<0.001)], for the delayed phase (24-120 hours), were 31.26±2.57, 67.91±2.12 ,[(55.11% reduction;(p<0.001)] respectively.

**Conclusion:**

Olanzapine, along with dexamethasone and granisetron, significantly reduced vomiting and nausea in patients undergoing chemotherapy. No adverse event of olanzapine was observed in the patients.

Nausea and vomiting are one of the serious side effects of chemotherapy that severely affect the quality of life of patients. It is known as major side effects from the patients' point of view (1-3). 5-HT3 receptor antagonists such as granisetron plus dexamethasone and aprepitant are the usual drugs prescribed for the prevention of chemotherapy-induced nausea and vomiting (CINV) in patients receiving moderate and high emetogenic anti-cancer drugs (1,3,4). Complete response (CR) for acute and delayed period in patients undergoing chemotherapy with the use of these drugs has been respectively 45-85 and 25-67 percentages (5, 6). Complications such as anorexia, malnutrition, dehydration, weakness, weight loss and electrolyte imbalances, result from CINV that can lead to poor compliance with subsequent chemotherapy cycles and increased anxiety towards treatment (2, 7). These cause patients to fear the next cycles of chemotherapy due to anxiety associated with bad experience (8). 

It is important that patients receiving chemotherapy for the first time, and they should be free of CINV as far as possible for increasing compliance in next treatments. Olanzapine is accepted by the Food and Drug Administration (FDA) as an antipsychotic drug. Potentially various neurotransmitters are inhibited by olanzapine. These include: serotonin at 5-HT2a, 5-HT2c, 5-HT3, and 5-HT6 receptors, dopamine at D1, D2, D3, and D4 receptors in the brain, catecholamines at alpha-1 adrenergic receptors, acetylcholine at muscarinic receptors, and histamine at H1 receptors (9). Since olanzapine has a comprehensive and powerful prohibiting activity at numerous receptors involved in nausea and vomiting pathways, this drug is a useful treatment for CINV (4). Its activity particularly at the D2, 5-HT2c, and 5-HT3 receptors, which connect with nausea and vomiting, discloses that it may have considerable antiemetic features (10). A phase II trial showed that olanzapine combined with dexamethasone and palonosetron, was very efficient at managing acute and delayed CINV (11). Another study reported no significant difference was observed between olanzapine and aprepitant in prohibiting CINV with extremely emetogenic chemotherapy drugs (8). 

According to literature, there are controversies in the control of CINV by olanzapine. To fill this gap, the purpose of this study was to compare the efficacy of olanzapine and placebo, each combined with granisetron and dexamethasone in preventing CINV in patients receiving high or moderate emetogenic drugs. According to the Functional Living Index Emesis (FLIE) scale, this study was conducted during three phases: overall phase (0-120 hours), acute phase (0-24 hours), and delayed phase (24-120 hours). To our knowledge, this is the first trial in Iran that investigated the effect of olanzapine on CINV in chemotherapy-naïve patients.

## Methods

**Study Design: **This trial was conducted between September 2017 and August 2018 in the Hematology-Oncology ward of Imam Khomeini Hospital in Urmia-Iran. The study sample included a consecutive group of chemotherapy naive patients treated during a 1-year period. The patients had to be planned for treatment with either high or moderate emetogenic chemotherapy drugs.

**Inclusion criteria:** Patients aged 18 years or older, with cancer who did not receive previous chemotherapy, receiving high or moderate emetogenic chemotherapy, hospitalized in ward, no nausea or vomiting during 24 hours before participating, had no known hypersensitivity to olanzapine; no known cardiac arrhythmia, congestive heart failure, or acute myocardial infarction within the previous six months and provided written and informed consent. 

**Exclusion criteria:** The patients that already received chemotherapy; were receiving chemotherapy combined with radiotherapy, had diabetes mellitus and hyperlipidemia, were undergoing treatment with antipsychotics. 

**Study procedure:** All patients qualified for the study were divided randomly into two groups of olanzapine, granisetron and dexamethasone (OGD) regimen and the placebo, granisetron and dexamethasone (PGD) regimen. This division was performed according to a computer-generated random assignment program created by a statistician who was not involved with the study. The patients were categorized according to gender and chemotherapy regimen (High or Moderate/Low emetogen drugs). All of the patients that received the OGD regimen had an antiemetic regimen consisting of dexamethasone 8 mg and granisetron 1mg three times daily on the first day of chemotherapy Also, they received olanzapine 5mg (B.W: 60kg or less) or 10 mg (B.W: more than 60kg) PO the day before chemotherapy and maintained it for 1–5 days pursuing the chemotherapy administration. The patients in PGD regimen received a placebo instead of olanzapine. 

**Randomization and blinding: **The patients were randomly assigned into two groups using a balanced randomization method. The patients and the investigators who did clinical assessments were blinded to the treatment groups and kinds of medication. The drug and the placebo were given to the patients in same packs and with the equal doses (containers had same weight and were similar in appearance).

**Outcomes:** The enrolled patients were hospitalized for the treatment the day before and six days after the start of chemotherapy. We recorded clinical data on each patient at the time of hospitalization. The efficacy of both regimens was assessed by FLIE scale which is a validated, -nausea and vomiting-specific, patient reported quality of life questionnaire consisting of 18 questions. It is consisted of two domains (vomiting and nausea) with nine similar items in every area. The score of the FLIE is specified by adding the answers to 18 questions on a 7-point analogue scale and, therefore, the possible range of total scores is between 18 and 126(12). The first end point, no nausea, no vomiting was described as a rate of patients without nausea and vomiting during the overall evaluation period (0 to 120 hours), the second endpoint was the mean scale of patients without nausea, and vomiting in the acute assessment period (0 to 24 hours)***, ***and the third endpoint was defined as no nausea, no vomiting in the delayed assessment period (25 to 120 hours) after chemotherapy was based on FLIE scale. 

**Statistical Analysis:** The data were analyzed by SPSS software Version 19.0. The patients' demographic data were summarized by descriptive statistics in FLIE data analysis. Chi-square and t-test were used to compare qualitative and quantitative data between the two groups. For comparing the Mean ±ED in the groups and in three- periods of study (acute, delayed and overall) the repeated measures ANOVA and Bonferroni post hoc test were used. A p- value of <.05 was considered statistically significant.

**Ethical considerations:** This study was conducted following the ethical principles (Declaration of Helsinki). The clinical protocol and all relevant documents were reviewed and approved by the Ethics Committee of Urmia University of Medical Sciences, with number: Ir.umsu.rec.1396.131 and was registered at the Iranian Registry of Clinical Trials (www.irct.ir) under the registration number of IRCT20170814035697N3. The privacy of the patients was respected by guaranteeing their anonymity. The main researcher kept the patients’ information and records of the procedure were strictly confidential, was made by the primary researcher in the respective rooms before treatment to clarify the risks and benefits as well as to address the answers to other questions or concerns. 

## Results

**Figure 1:**
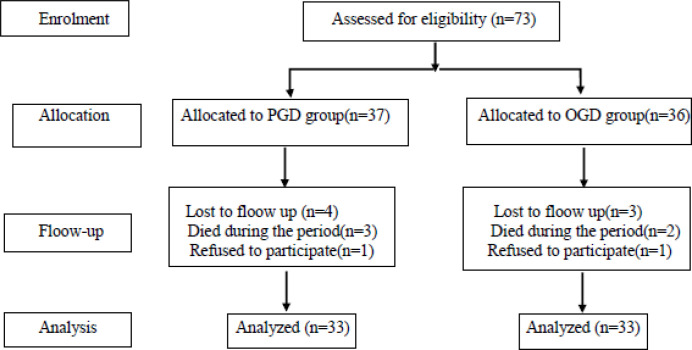
Study participants: Enrollment, allocation, follow-up and analysis

A total of 66 participants were enrolled in the study (fig. 1). Demographic and clinical data are shown in table-1. No significant differences were observed between the olanzapine and placebo groups in terms of age, sex, chemotherapy regimen administered, or type of cancer. 

The primary endpoint (no nausea, no vomiting) was 57.93% reduction compared to control group in overall phase. The secondary endpoint (no nausea, no vomiting) in acute phase was 47.21% and the third endpoint (no nausea, no vomiting) was 55.11% in delayed phase (p<0.001 for all phases). Other results are shown in Table 2. 

The results of the analysis of repeated measurement variance indicated that the main effect of time, the intervention (olanzapine), and the interaction effect of time with intervention were significant (p<0.001) (table 3). Post hoc analysis of olanzapine success rates by periods of treatment (acute, delayed and overall) in two groups by using Bonferroni test revealed that routine treatment (PGD) significantly reduces the total score of nausea and vomiting during the acute period and has a significant difference with the score of days 3 and 5 (p<0.001), but the score of days 3 and 5 had no significant difference (p> 0.05), that is, routine therapy and placebo in controlling nausea and vomiting were not successful in latency. In the OGD group, the response to olanzapine is almost the same in all three periods, and there is no significant difference between the three periods (p>0.05) (table 4).

**Table 1:** Patient demographic and disease characteristics

**Patient’s Characteristics**		**OLN group**		**Control group**		**Chi-square test**
		**%**	**N**	**%**	**N**	**P-value**
sex	male	51.52	17	66.67	22	0.87
	female	48.48	16	33.33	11	
Emetogenicity of chemotherapy drugs	H	36.4	12	36.4	12	0.952
	M	30.3	10	27.2	9	
	H+M	33.3	11	36.4	12	
type of cancer	GIT	6.1	2	6.1	2	
	Genital	15.2	5	6.1	2	
	ALL	42.4	14	48.4	16	
	AML	27.2	9	30.3	10	
	Multiple myeloma	6.1	2	6.1	2	
	Sarchoma	0	0	3	1	
	Respiratory	3	1	0	0	

H: Highly Emetogenic Chemotherapy, M: Moderately Emetogenic Chemotherapy, H+M: Highly +Moderately Emetogenic Chemotherapy, GIT: Gastric Intestinal Tract. ALL: Acute lymphocytic leukemia, AML: Acute myeloid Leukemia

**Table 2:** Results of the effect of olanzapine in comparison with placebo on the mean score of nausea, vomiting and nausea with vomiting in OGD and PGD groups

		Day 1	Day 3	Day 5
Nausea	Mean ± SE (PGD)	27.27±8.66	34.48±10.15	35.64±10.19
	Mean ± SE (OGD)	13.41±6.87	15.18±6.39	14.48±7.56
	Mean difference of 2 groups	13.87±1.74	19.30±3.86	21.13±2.68
	% reduction in nausea	50.21	55.79	59.14
	*P-value*	*<0.001*	*<0.001*	*<0.001*
Vomiting	Mean ± SE (PGD)	24.14±11.32	33.25±9.28	34.12±13.66
	Mean ± SE (OGD)	14.12±6.07	15.16±6.25	14.27±6.63
	Mean difference of 2 groups	10.22±2.22	17.92±1.6	18.77±2.68
	% reduction in vomiting	42.62	54.12	56.07
	*P-value*	*<0.001*	*<0.001*	*<0.001*
Nausea and Vomiting	Mean ± SE (PGD)	51.85±2.24	67.91±2.12	69.75±2.32
	Mean ± SE (OGD)	26.08±2.36	31.26±2.57	29.94±2.06
	Mean difference of 2 groups	25.58±4.69	37.81±3.43	39.81±4.34
	% reduction in nausea and vomiting	47.21	55.11	57.93
	*P-value*	*<0.001*	*<0.001*	*<0.001*

**Table 3:** Results of analysis of variance for nausea+vomiting

**Source**	**Sum of Squares**	**Degree of Freedom(df)**	**Meam Square**	**F**	**P-value**	**Partial Eta Squared**
Time	4357.2	2	2179.6	21.99	<0.001	0.24
Time * Intervention	2472.5	2	1236.7	12.5	<0.001	0.15
Error	13541	136	99.1			
Intervention(olanzapine)	59202.7	1	59203.9	89.6	<0.001	0.59
Error	40892.9	68	600.7			

**Table 4:** The rate of reduction in the total score of nausea and vomiting in 3 periods between groups

**group**	**Period(1,3,5)**	**Mean diference**	**Std.error**	**p- value**
PGD	1 vs3	16.16	1.89	<0.001
	1 vs 5	17.90	2.24	<0.001
	3 vs 5	1.84	1.69	1.00
OGD	1 vs 3	5.18	2.11	0.37
	1 vs 5	3.86	2.54	087
	3 vs 5	1.32	1.21	1.00

## Discussion

In this study, it was observed that olanzapine combined with dexamethasone and granisetron is more efficient in managing acute and delayed CINV. Our most important finding in this study was the use of three drugs instead of four drugs in complete control of nausea and vomiting in acute and delayed periods. Acute vomiting is the most important factor for poor vomiting prognosis (13). When vomiting occurs once, this is expected in subsequent chemotherapy (14). Therefore, in this study, patients in the first period of chemotherapy were used to eliminate the expected nausea and vomiting. An article that studied the effect of olanzapine compared to placebo in patients who received dexamethasone, aprepitant and a 5-HT3 antagonist (4) no nausea in the first 24 hours (acute phase), was 74% in the intervention group versus 45% in the placebo group. In our study, the reduction of nausea score was significant between the control and intervention groups also (p<0.001). In the same study, the complete response rate (no nausea and vomiting) was 42.4% versus 25.4% in the delayed phase (p<0.001). Our study also showed a significant reduction in nausea and vomiting score (p<0.001). We believe this outcome is valuable because we replaced an expensive drug (aprepitant) with a cheap drug (olanzapine). Another important finding in this study was that delayed nausea and vomiting decreased same as the acute phase in the OGD group. Although the prevention of delayed vomiting is more difficult than the acute type (1), our results showed that there was a significant difference between the mean ±SD in the first day with the third and the fifth day in the control group (p<0.001), but this difference was not significant in intervention group. These findings are according to results of Abe et al. (1) and Navari et al. studies (15). 

In another study (16), although the results were consistent with our results, the control of nausea and vomiting was less than our study. It seems that the reason for this difference was related to type of drugs of chemotherapy. Patients in that study received only high emetogenic drugs. However, in our study, about one third of the patients treated by high emetogenic drugs and two third received moderate and high emetogenic drugs. In a phase III clinical trial that patients received cisplatin (17, 18) or anthracycline and cyclophosphamide (19, 20), aprepitant was not more effective in reducing nausea. The other differences between the mentioned studies and the present study were where the patients in the intervention group in addition to olanzapine received three anti-vomiting drugs (aprepitant, dexamethasone, and an inhibitor of 5-HT3 receptors) that our patients did not receive aprepitant. Babu G et al. (8) have shown that there is not difference between aprepitant and olanzapine in the prevention of CINV. Since olanzapine is cheaper compared to aprepitant, it seems that the use of this drug is economical. 

The present and many other studies (1, 15, 16, 21) have shown that CINV is significantly reduced when olanzapine is added to routine drugs. The efficacy of olanzapine in controlling nausea is comparable with the findings of trials using aprepitant. Although aprepitant and its families (fosaprepitant, netupitant, rolapitant) controlled vomiting in acute and delayed phase, but it was less effective in controlling nausea (22). It seems that aprepitant’s efficiency may decrease in the presence of olanzapine. On the other hand, olanzapine acts as an antagonist for dopamine (D1-D5), serotonin (5-HT2a, 5-HT2c, 5-HT3, 5-HT6) histamine (H1), adrenaline (α1) and acetylcholine (m1-m5) receptors (23). H1 and Ach (M) receptors are found in the vestibular apparatus, that cause nausea. Aprepitant cannot penetrate to this apparatus but olanzapine can (24), though the effect of olanzapine in controlling nausea is more than aprepitant. This finding has been shown in Navari RM et al.’s study also (10). 

Another important finding in the present study was that during treatment, no adverse events were observed in patients receiving olanzapine but in other similar studies drowsiness was reported in some patients (4, 8, 15). A limitation of our study was that we evaluated all type of cancers together. Since treatment regimens are different for each type of cancer and have different severity of nausea and vomiting (high, moderate or low), so it is best to study only one treatment regimen or one type of cancer. Due to patient limitations, we did not perform this distinction. 

In conclusion the results of this clinical trial showed that olanzapine, along with dexamethasone and granisetron, in patients undergoing chemotherapy with HEC and MEC drugs or combination of both, CINV were significantly lower than the patients who did not receive olanzapine. No adverse event of olanzapine was observed in patients undergoing treatment.

## References

[1] Abe M, Hirashima Y, Kasamatsu Y (2015). Efficacy and safety of olanzapine combined with aprepitant, palonosetron, and dexamethasone for preventing nausea and vomiting induced by cisplatin-based chemotherapy in gynecological cancer: KCOG-G1301 phase II trial. Support Care Cancer.

[2] Navari RM (2015). 5-HT3 receptors as important mediators of nausea and vomiting due to chemotherapy. Biochim Biophys Acta.

[3] Nikbakhsh N, Sadeghi MV, Ramzani E (2016). Efficacy of olanzapine in symptom relief and quality of life in gastric cancer patients receiving chemotherapy. J Res Med Sci.

[4] Navari RM, Quin R, Ruddy KJ (2016). Olanzapine for the prevention of chemotherapy-induced nausea and vomiting. N Engl J Med.

[5] Chiu L, Chiu N, Chow R, Zhang L (2016). Olanzapine for the prophylaxis and rescue of chemotherapy-induced nausea and vomiting (CINV): a retrospective study. Ann Palliat Med.

[6] Janelsins MC, Tejani M, Kamen C (2013). Current pharmacotherapy for chemotherapy-induced nausea and vomiting in cancer patients. Expert Opin Pharmacother.

[7] Wang XF, Feng Y, Chen Y, Gao BL, Han BH (2014). A meta-analysis of olanzapine for the prevention of chemotherapy-induced nausea and vomiting. Sci Rep.

[8] Babu G, Saldanha SC, Chinnagiriyappa LK, Jacob LA, Mallekavu SB, Dasappa L (2016). The Efficacy, safety, and cost benefit of olanzapine versus aprepitant in highly emetogenic chemotherapy: a pilot study from South India. Chemother Res Pract.

[9] Tan L, Liu J, Liu X (2009). Clinical research of Olanzapine for prevention of chemotherapy-induced nausea and vomiting. J Exp Clin Cancer Res.

[10] Navari RM, Gray SE, Kerr AC (2011). Olanzapine Versus Aprepitant for the prevention of chemotherapy-induced nausea and vomiting: a randomized phase III trial. J Support Oncol.

[11] Navari RM, Einhorn LH, Loehrer PJ Sr (2007). A phase II trial of olanzapine, dexamethasone, and palonosetron for the prevention for the prevention of chemotherapy-induced nausea and vomiting, Support. Care Cancer.

[12] Aksu G, Dolaşık I, Ensaroğlu F (2013). Evaluation of the efficacy of aprepitant on the prevention of chemotherapy-induced nausea and vomiting and quality of life with functional living index emesis. Balkan Med J.

[13] Italian Group for Antiemetic Research (2000). Prevention of cisplatin-induced delayed emesis: still unsatisfactory. Support Care Cancer.

[14] Roscoe JA, Morrow GR, Aapro MS, Molassiotis A, Olver I (2011). Anticipatory nausea and vomiting. Support Care Cancer.

[15] Navari RM, Nagy CK, Le-Rademacher J, Loprinzi CL (2016). Olanzapine versus fosaprepitant for the prevention of concurrent chemotherapy radiotherapy-induced nausea and vomiting. J Community Support Oncol.

[16] Navari RM, Quin R, Ruddy KJ (2015). Olanzapine for the prevention of chemotherapy-induced nausea and vomiting (CINV) in patients receiving highly emetogenic chemotherapy (HEC): Alliance A221301 a randomized double-blind, placebo-controlled trial. J Clin Oncol.

[17] Hesketh PJ, Grunberg SM, Gralla RJ (2003). The oral neurokinin-1 antagonist aprepitant for the prevention of chemotherapy-induced nausea and vomiting: a multinational, randomized, double blind placebo-controlled trial in patients receiving high-dose cisplatin. J Clin Oncol.

[18] Hesketh PJ, Grunberg SM, Herrstedt J (2006). Combined data from two phase III trials of the NK-1 antagonist aprepitant plus a 5HT3 antagonist and a corticosteroid for prevention of chemotherapy-induced nausea and vomiting: effect of gender on treatment response. Support Care Cancer.

[19] Rapoport BL, Jordan K, Boice JA (2010). Aprepitant for the prevention of chemotherapy-induced nausea and vomiting associated with a broad range of moderately emetogenic chemotherapies and tumor types: a randomized, double-blind study. Support Care Cancer.

[20] Warr DG, Hesketh PJ, Gralla RJ (2005). Efficacy and tolerability of aprepitant for the prevention of chemotherapy-induced nausea and vomiting in patients with breast cancer after moderately emetogenic chemotherapy. J Clin Oncol.

[21] Vig S, Seibert L, Green MR (2014). Olanzapine is effective for refractory chemotherapy-induced nausea and vomiting irrespective of chemotherapy emetogenicity. J Cancer Res Clin Oncol.

[22] Navari RM (2015). Rolapitant for the treatment of chemotherapy-induced nausea and vomiting. Expert Rev Anticancer Ther.

[23] Kast RE, Foley KF (2007). Cancer chemotherapy and cachexia: mirtazapine and olanzapine are 5-HT3 antagonists with good anti-nausea effects. Eur J Cancer Care.

[24] Clemmons AB, Orr J, Andrick B (2018). Randomized, Placebo-Controlled, Phase III Trial of Fosaprepitant, Ondansetron, Dexamethasone (FOND) Versus FOND Plus Olanzapine (FOND-O) for the prevention of chemotherapy-induced nausea and vomiting in patients with hematologic malignancies receiving highly emetogenic chemotherapy and hematopoietic cell transplantation regimens: The FOND-O Trial. Biol Blood Marrow Transplant.

